# A CTSA One Health Alliance guidance on institutional review of veterinary clinical studies

**DOI:** 10.1186/s12917-021-02790-4

**Published:** 2021-02-17

**Authors:** S. A. Moore, A. O’Kell, H. Borghese, R. Garabed, H. O’Meara, P. Baneux, CTSA One Health Alliance

**Affiliations:** 1 Department of Veterinary Clinical Sciences, The Ohio State University College of Veterinary Medicine, 601 Vernon L Tharp St, Columbus, OH 43210 USA; 2 Department of Small Animal Clinical Sciences, University of Florida College of Veterinary Medicine, Gainesville, USA; 3 The Ohio State University College of Veterinary Medicine, Blue Buffalo Veterinary Clinical Trials Office, Columbus, USA; 4 Department of Veterinary Preventive Medicine, The Ohio State University College of Veterinary Medicine, Columbus, USA; 5 The Ohio State University, Office of Responsible Research Practices, Columbus, USA; 6 Cornell University, Attending veterinarian, Director Center for Animal Resources and Education, Ithaca, USA

## Abstract

Harmonized institutional processes and reviewer training are vital to maintain integrity and ethical rigor of the veterinary clinical research pipeline and are a prerequisite to future work that might establish centralized or single-site ethical and regulatory review to ease initiation of multi-center studies. Funded by a CTSA One Health Alliance (COHA) pilot award, a diverse working group of veterinary clinicians and institutional representatives was convened in February 2020 to develop a guidance document detailing broadly agreed upon practices for ethical review and approval of veterinary clinical studies conducted in the United States.

The working group defined key areas of need for consensus, developed a set of associated guidelines, and circulated these for review by COHA’s fifteen member institutions. Six focus areas were identified by the working group and included vital items of protocol review, composition of the review committee, post-approval monitoring and adverse event reporting, consideration of special circumstances such as satellite sites and the use of healthy veterinary subjects in research, and the informed consent process.

This document outlines a broadly agreed-upon framework through which to approach vital items associated with veterinary clinical study protocol review and approval. These approaches represent current best practice in the review and approval of veterinary clinical studies, and can serve as a guidance for veterinary clinician-scientists and regulatory experts, to ensure robust and ethically conducted studies that can contribute to the advancement of both animal and human health.

## Background

With increasing collaborative initiatives across the landscape of human and veterinary medicine, a unique opportunity exists for a comparative medicine approach that leverages natural disease in veterinary patients to positively impact translational medicine efforts such that both animals and people benefit. An existing major impediment to more broadly engaging the scientific community in veterinary clinical studies is the lack of clear guidance regarding oversight of research in client-owned animals. While reduced regulatory burden in the veterinary clinical setting can enhance the pace of science, a lack of guidance and universal protocols opens the veterinary community to scrutiny, particularly when adverse outcomes occur in animals enrolled in clinical studies. Moreover, the absence of standard processes and procedures has created an environment in which individual institutions have established site-specific processes for approval and monitoring of clinical studies making harmonizing cross-institutional efforts challenging. Largely exempt from United States (US) federal laws such as the Animal Welfare Act, which was developed to provide standards for the use of animals in laboratory research, ethical considerations relevant to the review and approval of veterinary clinical studies now more closely resemble those of human clinical trials [1]. While institutional review boards (IRB) have a federal mandate to guide review of human trials, there is no clear guidance on what the review process should look like for veterinary clinical studies.

Uniform processes and reviewer training are vital to maintain integrity and ethical rigor of the veterinary clinical research pipeline and are a prerequisite to future work that might establish centralized or single-site review to ease initiation of multi-center studies. The CTSA One Health Alliance (COHA) is a US-based group of academic veterinary medical centers partnered with clinical and translational researchers through NIH-administered Clinical and Translational Science Awards (CTSA). The overarching mission of COHA is to advance understanding of diseases shared by humans and animals. Of particular interest is the value of veterinary clinical studies to contribute to bidirectional advancement in health research. An important focus for the group is the development and refinement of infrastructure and processes that enhance the efficiency, rigor, and reproducibility of veterinary clinical studies conducted across the US.

Funded by a COHA pilot award, a diverse working group of veterinary clinicians and institutional representatives was convened in February 2020 to develop a guidance document detailing broadly agreed upon practices for ethical review and approval of veterinary clinical studies. The working group defined key areas of need for consensus, developed a set of associated guidelines, and circulated these for review by COHA’s fifteen member institutions. The document that follows represents the outcome of that process.

While several authors have previously published on institutional review and approval processes for veterinary clinical study protocols, there are no uniformly accepted standards for a harmonized approach across institutions [2, 3]. The goal of this document is to outline a framework for best practice in the review and approval of veterinary clinical studies, as agreed upon by a broad network of veterinary clinician-scientists and regulatory experts, to ensure robust and ethically conducted studies that can contribute to the advancement of both animal and human health. While some aspects of this document are specific to US regulatory requirements and institutional perspectives, many aspects address broader concepts of ethical trial conduct that can be applied regardless of geographic location.

### Unique ethical considerations for veterinary clinical studies

Signed in to law in 1966, the Animal Welfare Act was originally intended to prevent the theft and subsequent sale of privately owned animals to research facilities and was later expanded to “regulated groups and individuals” that “exhibit, transport, or use animals in the pursuit of medical and scientific knowledge” (https://awahistory.nal.usda.gov/timeline). The Animal Welfare Act is administered by the United States Department of Agriculture (USDA) and outlines minimum acceptable standards for the treatment of animals bred for use in research (https://www.nal.usda.gov/awic/animal-welfare-act). In 1985, the act was amended to require the formation of institutional animal care and use committees (IACUC) to review studies that proposed to use animals for research and provided guidance on the reduction of pain and distress in laboratory animals.

One of the foundational premises of the Animal Welfare Act is that laboratory animals do not have an inherent advocate and thus federal regulations must guide their ethical use in research. This is, of course, not the case with client-owned animals in the context of veterinary clinical studies; however, federal funding sources still mandate IACUC review for studies involving client-owned animals [4]. Unique to veterinary clinical studies, animals are presented by their owner/care-givers, who are seeking research involvement in the context of veterinary health care and bring an additional set of ethical considerations that extend beyond treatment of the animal itself. These include the existence of the human/animal bond and its influence on willingness to enroll a pet in a clinical study; the veterinary client-patient relationship and the potential for lines between clinical caregiver and researcher to be blurred; differing endpoints and early study removal criteria than may be allowable or relevant for research using purpose-bred animals; and the concept of informed consent for owners and how that may differ when the study subject is an individual animal versus a group or herd of animals.

It is clear that groups reviewing veterinary clinical studies must consider some aspects of the regulatory framework surrounding the use of animals in research, but they also must examine ethical questions similar to those articulated by the National Institutes of Health (NIH) as vital during human trial review and approval as well as have a clear understanding of the practice of and regulations associated with veterinary medicine. This means that robust and efficient review and approval of veterinary clinical studies requires an understanding of complex regulatory, scientific, and hospital/ethical issues. Therefore, they must involve a collaborative approach between individuals experienced in clinical research (IRB-like evaluation) and veterinary medical practice as well as IACUC representatives with a broad understanding of federal regulatory requirements and guidance as well as animal welfare oversight [2, 3].

### Establishing a framework for ethical review and approval

The fundamental goal of clinical studies is improvement in health or advancement in common knowledge. Research protocols must be methodically rigorous and practically feasible to accomplish these goals. Currently, the governing review body for veterinary clinical studies varies by institution, typically involving an IACUC, a veterinary clinical studies committee, or sometimes a parallel process involving both committee types [2, 3]. For the purposes of discussion, we will refer to these committees collectively as veterinary review boards. These groups currently review study protocols for value and validity, but processes differ widely across institutions based on committee composition, background and objectives.

While it is acknowledged that the process for review and approval of veterinary clinical studies may differ by institution, recommendations made within this guidance document are intended to provide a checklist to ensure that all vital aspects of review are covered at an individual institution. They can also serve as a springboard for discussion in areas where further refinement is needed. For the purposes of this document, we have adopted the definition used by the American Veterinary Medical Association (AVMA): “a veterinary clinical study involves research that gains information from animal patients. Clinical studies are intended to advance animal health care by identifying the most effective therapies and practices for a given condition, or by advancing our basic understanding of the disease” (https://ebusiness.avma.org/aahsd/more_info/veterinary_clinical_studies.aspx). The AVMA further indicates that clinical trials/interventional studies as well as observational studies are included in this definition. Six focus areas were identified by the working group and included vital items of protocol review, composition of the review committee, post-approval monitoring and adverse event reporting, consideration of special circumstances such as satellite sites and the use of healthy veterinary subjects in research, and the informed consent process. Important considerations for each area are discussed and opportunities for the development of additional resources and guidelines are identified.

### Vital review items

Establishing a set of defined elements that are evaluated by every review board prior to approval of a veterinary clinical study protocol represents the first step toward a harmonized approach across veterinary medical centers. Items identified by the working group as vital aspects of the review process for veterinary clinical studies are summarized in Table 1, with key elements discussed in detail below.

**Table 1:** Recommended items for consideration during review and approval of veterinary clinical studies. Associated with each review item are several questions the committee may ask themselves as they review a proposed study

Review item	Associated questions
Risk/benefit	Does the individual patient stand to benefit? Is the potential for benefit proportional to potential for harm? Are risks clearly defined, balanced, and mitigated?
Societal value	In the absence of direct patient benefit, is the benefit to the veterinary or translational science community at least clearly defined and proportional to potential harm?
Scientific validity	Is the scientific approach justified by the study team? Has the protocol had previous scientific merit review? Are inclusion/exclusion criteria clearly stated and appropriate? Are outcomes relevant and justified? Is proposed sample size appropriately justified?
Infrastructure	Is the study team qualified to conduct the proposed work? Are infrastructure and staffing adequate to support the project? Does hospital or regional caseload support feasibility? Will other competing studies affect enrollment?
Biosafety considerations	Are animal, study team, owner, and environmental risks appropriately mitigated? Are all institutional biosafety approvals in place?
The consent process	Is the consent form structured to enhance readability and understanding? Are risks and benefits clearly defined to the owner? Are adverse event descriptions current based on accumulating data? Does the form and process permit withdrawal of consent? Is the consent form congruent with protocol and study design? Are financial requirements of, and compensation to the owner, including adverse event coverage, clearly articulated? Are future, yet undefined, uses of samples or data intended? If so, is this addressed? Is there any aspect of the consent process (hierarchical relationship, undue inducement, etc.) that would diminish the owner’s agency in consenting for their animal?
Adverse event reporting	Are procedures for reporting clearly defined? To whom will minor versus serious adverse events be reported and with what frequency? How will reporting across sites be handled? What criteria will determine if a patient is removed from the study? What criteria might necessitate the study being halted entirely?
Conflict of interest	Does a conflict of interest exist for any member of the study team? Does a conflict of interest exist with the funding source? Is a conflict management plan in place?
Incentives	Are incentives reasonable with consideration to avoid undue inducement?
Other considerations	Is privacy/confidentiality protected as required by legal and institutional guidelines? Are datasets and data risks managed per institutional policy?

#### Risk/benefit

Of primary consideration during review of veterinary clinical studies should be the balance of risk and benefit, with the understanding that the relationship between the two is a sliding scale such that as risk increases, so too must potential for benefit to the veterinary patient. Specifically, review boards might approve a study that involves more than minimal risk to a patient if the intervention has the potential to provide a direct benefit to that patient and the risk presented is balanced by the degree and anticipation of benefit [5, 6]. This crucial balancing of benefit and risk is an inherently subjective matter and therefore requires broad input from constituents of the review board in establishing norms and thresholds for what is considered appropriate risk. In evaluating risk, it is important to distinguish the difference between inherent risk associated with diagnosis and treatment of the animal’s condition and risk associated with enrollment in the clinical study. The process by which risk is assessed, in relation to benefit, should be defined locally, and future production of training resources that facilitate risk assessment in veterinary clinical studies represent an opportunity for refinement of this process.

During IRB review of human subjects research, risk assessment is often accomplished by a process called “component analysis”, where each activity associated with a study that extends beyond routine intervention is evaluated to assess whether it is being performed with therapeutic intent or solely for data collection purposes. For each element of the study, the level of risk is evaluated to ensure that it has been appropriately minimized. Potential for harm to subjects should be proportional to potential for benefit. High risk activities that do not have the “prospect of direct benefit” deserve more scrutiny [5]. This is often encountered on the human side in the phase I, or “first in man”, clinical trials where direct benefit is not the purpose of the study nor is it anticipated in most instances. In these scenarios, rationale for approval lies in balancing the risk to the patient with the overall potential benefit to society as a whole, often termed “contribution to science” or “societal value” [7].

#### Societal value

In human subject research, in the absence of direct health benefit to human patients, the risk of study enrollment may be balanced, at a minimum, by the potential for overall value to society, assuming consent is voluntary and informed. In such scenarios, the concept of “risk/benefit” assessment may be replaced by a “risk/value” assessment [7, 8]. A similar approach can also be used in evaluating veterinary clinical studies with low or no anticipated patient benefit. The concept of societal value is, however, a somewhat ambiguous term, and review of societal value requires members of the review board to make judgements based on their background, training and experience.

A handful of recent publications carefully explore the concept of societal value in the context of the human clinical research setting [9, 10]. To the authors’ knowledge, no publications evaluate this concept thoroughly in the context of veterinary clinical research. Therefore, an approach for how societal value is weighted by veterinary ethical review boards must be drawn from the human medical literature with consideration for some unique differences. Assessment should include evaluation of whether the proposed research balances the lack of direct patient benefit with overall potential for a benefit to the veterinary community that is clearly articulated both within the study protocol and in the written consent form.

An additional inherent challenge to evaluating this aspect of some veterinary clinical studies is that the ultimate intention may not be to develop a treatment for use in veterinary medicine, rather the study may be evaluating a therapy ultimately intended only for the human market. This issue is becoming increasingly important as questions regarding the relevance of preclinical rodent studies escalate and the desire for preclinical models of disease that more accurately represent the genetic and environmental variability present in human disease increases [11]. Given the original focus of the Animal Welfare Act on preventing the use of pets in human medical research without owner consent, studies without direct benefit to the veterinary community require careful evaluation. In these studies, the risk/benefit to the individual animal should be of utmost consideration due to the lack of societal value to the veterinary community. However, it should also be noted that the use of extra-label drugs is commonplace in veterinary medicine in some countries, including the United States (https://www.avma.org/extralabel-drug-use-and-amduca-faq), and the information gained from studies designed for human medical translational therapies might still benefit future veterinary patients although potential cost and feasibility might warrant consideration. The involvement of healthy animals in some early stages of therapeutic development adds additional complexity and is discussed in depth as a special issue below.

#### Scientific validity

The question of whether review boards should assess the scientific merit of a proposed study can serve as a point of confusion for reviewers and as a cause for animosity between clinical investigators and review boards [12–14]. Some investigators argue that scientific review by ethics boards can lead to unnecessary hurdles to study initiation as protocols may have already been independently peer-reviewed and approved by content experts during the funding process [15]; however, particularly in veterinary medicine, the extent of scientific review varies considerably with the funding body. As such, the rigor with which a veterinary review board undertakes scientific evaluation of a protocol may vary depending on what previous review has already been performed. Additionally, some investigators argue against scientific evaluation during institutional review due to the limited probability that an ethical review board will have a content expert in the subject domain of their particular study, thus calling into question their ability to critically evaluate the science. A counter argument to this is that having someone removed from the particular field of study can be helpful in navigating bias and conflicting interests that someone entrenched in the field may overlook. Using this logic, one might argue that non-experts should be involved in the review of scientific merit of a protocol, as they are least likely to be biased or overestimate the potential patient benefit or societal value of a study [12]. Indeed, there are some well-noted examples of ethics committees having spotted substantial flaws in methodology not identified by scientific investigators [16].

Similar to the subjects of risk, benefit, and societal value, little to no guidance exists specific to the assessment of scientific validity in the context of veterinary clinical studies; however, review boards can draw from guidelines published for human trials. Basic assessment of scientific validity should be considered during the review of veterinary clinical studies, particularly in cases where rigorous scientific review has not been performed by the funding agency. Important considerations in this area should include whether the study has well-defined inclusion and exclusion criteria, whether the investigators have justified their selection of proposed outcomes, whether the sample size they propose is sufficiently supported based on existing literature or preliminary data relevant to the proposed work and whether appropriate local caseload and infrastructure are in place to feasibly conduct the activities proposed in the study protocol.

#### Use of incentives

Veterinary review boards should evaluate the nature of incentives provided to owners for research participation. An “incentive” is something that motivates or encourages someone to do something. When used in the context of clinical research, it is defined as anything that encourages someone to enroll or continue in a study [17]. Incentives in clinical research can take a number of forms (Table 2) [17]. The use of incentives to assist with clinical study recruitment and retention is common in both human and veterinary medicine and has been shown to improve study retention, with monetary incentives noted to be particularly effective [18]. One concern relating to the use of incentives is the concept of “coercion”, which refers to the denial of autonomy and consists of “... the deliberate imposition of one’s personal will on another … coercion usually takes the form of threats, which restrict people’s options” [19].

**Table 2:** Types of incentives used in veterinary clinical study recruitment and retention

Type of incentive	Example
Reimbursement for expenses incurred	Payment for an owner to travel to hospital visits associated with the research study, costs of screening tests or other medical expenses
Incentives to encourage a desired behavior	Prize lottery for completing an online questionnaire
Cash or cash-like rewards	Vouchers, money, gift cards
Social, emotional or tokenistic rewards	Donation to a charity, gift

In general, concerns surrounding incentives for clinical study enrollment would not fall into the category of coercion but might be more appropriately framed as an issue of “undue inducement”. Undue inducement describes a scenario where a person makes a choice, due to external factors, that might differ from the choice they would make based solely on intrinsic motivation [20]. Ethical concern over the use of incentives center on the idea that excessive incentivization of a trial may cross the line into “undue inducement” for research participation by overshadowing any intrinsic motivation the owner may have for enrolling in a study. This can become of particular concern in veterinary medicine, where most owners pay out of pocket for care. Subsidization of standard treatment, standard diagnostics, experimental therapy, experimental diagnostics, and enhanced monitoring are all potentially included as a collateral benefit to study enrollment. In this context, enrolling in a clinical study can allow a pet to receive care their owner might not otherwise be able to afford and therefore treatment subsidies may be factored by the owner when performing their own risk/benefit analysis prior to study enrollment. In the case where a beloved animal is experiencing a life-threatening condition, the offer of low-cost or free therapy might represent undue inducement if study participation is perceived as the only way to obtain treatment. Accordingly, an additional argument made against incentives is that owners will be more likely to be persuaded to subject their pets to risk when incentives are involved [12]. Alternatively, withholding incentives might prevent owners of lower socioeconomic status from participating in clinical studies and receiving its collateral benefits or might subject them to undue personal financial burden [21].

Clearly the issue of incentives in veterinary clinical research is complicated. Paying subjects for their participation in clinical research is, in most cases, considered an acceptable practice [13]. The question of incentives can present a challenge for review boards evaluating veterinary clinical studies, with a common question of “how much is too much”? The answer to this question is likely contextual, and dependent upon an assortment of study-related factors including the burden placed by the study on the owner and pet, the disease and intervention being studied, and the potential health benefits associated with the study. While not specifically addressed in the veterinary literature, an assortment of publications address this concept and evaluate the level of influence induced by financial incentives in human subjects research. These resources give particular consideration to socioeconomic status and severity of the research subject’s disease. Studies evaluating decision making in healthy volunteers suggest that moderate incentivization of trials, while potentially influencing whether or not a person enrolls in a study, does not cloud their ability to assess personal risk associated with enrollment [22–24].

There is clear guidance on the human side that incentives should not be evaluated by review boards as part of their risk/benefit analysis and rather should be considered as an entirely separate entity [13]. The office of Good Clinical Practice within the Federal Drug Administration specifically addresses incentives by noting that reimbursement for costs incurred in association with travel to participate in research are not considered undue inducements [13]. While in the context of human trials this typically includes things like airfare and lodging, one additional consideration in veterinary medicine is the requirement for owners to take time off work to present their pets for treatment and the potential for associated lost wages since sick leave may not be applicable in this scenario.

Ultimately, veterinary review boards should evaluate the type and quantity of incentives employed in a study to ensure that they balance the amount of time an owner will spend on research activities for their animal; travel costs; and any special considerations surrounding recruitment issues. Beyond that, additional guidance should be developed at the institutional level for what is allowable and appropriate while adhering to institutional policies and accounting requirements.

#### The consent process

The provision of voluntary informed consent, and the ability to withdraw that consent at any point during the study, is considered absolutely essential in veterinary clinical studies, and the process by which this is documented must be reviewed [2, 3]. Unlike most human clinical studies, veterinary clinical studies by definition involve a patient population that cannot provide informed consent or assent to risk associated with a study in the face of uncertain benefit. In this context, informed consent is given by the owner/care-giver of the animal. The COHA Clinical Studies Subcommittee has previously developed an informed consent template for use in veterinary clinical studies (https://www.ctsaonehealthalliance.org/resources/coha-informed-consent-template). Referencing this document may be useful for review boards to ensure that all key elements of consent are covered in written form by the investigators as they prepare consent documents specific to their study. Veterinary review boards should ensure that consent forms prepared by the investigator are congruent with their study design and protocol. For any studies offering no benefit to the animal, this should be clearly articulated in the consent form. Possible or anticipated benefits should be carefully described as direct or collateral benefit, and the potential benefits description should include the nature, magnitude, and likelihood of such benefits [21].

Bioethics literature supports the fact that the process of obtaining informed consent extends beyond the written consent form to include discussions with the study team and patient/owner that are influenced by context, presentation, and an individual’s cultural background and health literacy. Few veterinary-specific studies address the consenting process in clinical studies [25]; however, discussions focusing on best practices in consenting in veterinary clinical studies may draw in part from the pediatric literature, given some similarities related to the caregiver-provider scenario. Several recent studies have evaluated the complexities of pediatric informed consent and have identified common themes, all of which may have relevance to the veterinary consenting process. These include the influence of the doctor- patient [client] relationship, caregiver distress, caregiver comprehension (or lack thereof) of medical terminology and the study itself, and caregivers not being offered alternative treatments to the clinical trial [26]. Additionally, aspects of the consent process (hierarchical relationship, undue inducement, etc.) that would diminish the owner’s agency in consenting for their animal should be considered. In human-subjects research, the concept of undue inducement is not well defined. A national survey of IRB members and professionals involved in human-subjects research found variable levels of concern about offering financial compensation, non-monetary payment, or medical care to research patients [27]. The survey indicated that participants were more likely to consider reimbursement of expenses or compensation for time as being acceptable rather than offering money as an incentive to participate or to compensate for risk. These views were, however, inconsistent and the study concluded that further guidance in these areas is needed. Subordinate members of a hierarchal relationship are considered a vulnerable population in human-subjects research and are potentially subject to over-recruitment and/or compromise of the voluntary component of the consent process [28]. Suggestions to help alleviate this concern include the ability of the participant to discuss the research study with a third party not involved in the research, having an individual independent from the investigator obtain informed consent, and to stress the voluntary nature of the study [29] .There are no published veterinary studies addressing these issues, but the same concerns are likely to be relevant in veterinary clinical studies. Best practice in review and approval of veterinary clinical study protocols would include documentation that these issues are considered.

Perhaps a uniquely veterinary consideration in the context of informed consent is the scenario where a single owner or entity may consent for enrollment of a population of animals into a clinical study. In this case, a letter of support to the investigator or memorandum of understanding detailing the study protocol and intended use of the animals may be more appropriate than a consent form allowing enrollment of each individual animal.

#### Infrastructure

The veterinary review board should evaluate whether there is evidence that the proposed research team has adequate experience and training to complete the activities associated with the study. The study team should include individuals likely to have access to and be successful with enrollment of the intended veterinary patient population in order to ensure efficient completion of the study. Study team training related to best practices in consenting and other clinical research concepts should also be a consideration; though, documentation of this training may be variable. Additional items that might be considered by the review board include hospital caseload for the population of interest, whether adequate staffing exists to assist with study-related activities, and the presence of other competing studies in the hospital or in the region.

#### Adverse event identification, management, and reporting

Review of proposed studies should ensure that there is a documented plan for the identification, management, and reporting of adverse events associated with the study. A more in-depth discussion of how and when adverse events should be reported and reviewed is discussed below related to post-approval monitoring procedures. Assessment of quality of life factors during study participation is also highly relevant for many veterinary disease processes, and inclusion of these types of measures should be considered where relevant. For some veterinary patients, issues associated with diminished quality of life may be just as likely as an adverse event to impact whether they move off study [30].

#### Other items

Review of proposed studies should include evaluation of other items such as conflict of interest, management of data safety risks, and biosafety considerations. For some institutions, review of these items will fall to other bodies beyond the one (s) responsible for veterinary clinical studies review and approval. Additionally, most institutions will have their own guidelines and requirements for these factors. Veterinary review boards are encouraged to consult, or work collaboratively with, their local regulations, and other institutional review processes to ensure studies adhere to these, prior to study initiation.

### Review committee composition

The AVMA Veterinary Clinical Studies Committee states that veterinary clinical study review committees “should be composed of veterinarians primarily involved in clinical practice, should work closely with the IACUC, and have at least one member who is a member of the IACUC to serve as a conduit between the two entities” (https://www.avma.org/resources-tools/avma-policies/establishment-and-use-veterinary-clinical-studies-committees). In addition, each of the components of the review process discussed above should be able to be thoroughly evaluated by at least one member of the review board. Items identified by the working group for review, in broad terms, fell into several areas:

- Scientific, clinical, or societal validity
- Regulatory requirements
- Infrastructure/support requirements
- Computational validity

As such, committee composition should be tailored to address these areas by including a set of standing members as part of the review board. It is noted that addition of ad hoc members may be required to address questions or challenges associated with review of individual protocols. The recommended complement of review board members necessary to evaluate veterinary clinical studies is summarized in Table 3.

**Table 3:** Recommended committee composition for review and approval of veterinary clinical studies. While all members are encouraged to provide feedback on the entire protocol, they should provide, at a minimum, specific and formative feedback in their area of expertise. Committee size and composition will vary by institution and some members may cover more than one expert domain

Member	Specific review items
Scientific reviewer	Assessment of scientific validity and investigator justification for study design. This member need not be a content expert related to the protocol but rather should have familiarity with basic scientific study design and research conduct.
Clinical reviewer	Assessment of clinical validity, ethics and investigator justification for clinical study design. This member need not be a content expert related to the protocol but rather should have familiarity with basic clinical research design and conduct as well as applicability of the study to veterinary medicine at large.
Clinical study coordinator or equivalent	Assessment of infrastructure, caseload and other aspects of institutional feasibility.
Administrator	Assessment of infrastructure, caseload and other aspects of institutional feasibility.
IACUC member	Assessment of ethics and compliance with all relevant institutional and federal requirements for use of animals in research.
Quantitative evaluator	Assessment of sample size calculations, proposed treatment effects and overall study design.
Lay evaluator	Representation of community perspective on the proposed research with particular focus on readability of consent forms. Unlike federal guidelines for human subjects research review, there is no stipulation that this member be unaffiliated with the institution. Rather, they should simply represent a non-medical perspective on the presentation and conduct of the research.
Ad hoc members	Additional ad hoc members may be required for assessment of items such as biosafety, complex ethical or legal issues, particular scientific questions or conflicts of interest as such cases arise.

### Post-approval monitoring

Post-approval monitoring of veterinary clinical studies is necessary to ensure that studies are being conducted as approved and remain compliant with institutional and (where applicable) federal guidelines for use of animals in research. Recommended aspects of post-approval monitoring include a yearly review of protocol activities and ongoing reporting and evaluation of adverse events as they arise.

#### Continuing review

Post-approval monitoring should occur yearly and evaluate the following information: number of cases enrolled cumulatively to date and during the previous year, changes to the study team, a summary report of minor and serious adverse events provided by the investigator, a review of the consent form and a confirmation from the investigators that data storage adheres to institutional data management policies. For those studies including satellite locations, this review should also include an assurance from the principal investigator that case enrollment numbers have been reported, adverse events occurring at satellite locations have been conveyed, and consent forms are being properly maintained.

#### Adverse event reporting

Serious adverse events should be reported immediately to the review board for evaluation. The Veterinary Cooperative Oncology Group (VCOG) has developed a document outlining standard terminology for adverse event reporting in the context of veterinary oncology studies [17]. Most aspects of this document are broadly applicable across specialties and can serve as a foundation for a standard set of guidelines and terminology around adverse event reporting. Additionally, the COHA Clinical Studies Subcommittee has previously developed an adverse event reporting form for use in veterinary clinical studies (https://www.ctsaonehealthalliance.org/resources/coha-serious-adverse-event-sae-reporting-template). Attribution of the event as related or unrelated to the study intervention should be discussed by the study team and the veterinary review board. When a serious adverse event is reported, important considerations for both the review board and the investigator include whether the event should trigger any changes in the study consent form, warrants a protocol change, or is severe enough to halt a study (either temporarily or permanently). A particular consideration during review and approval of multi-site studies is the method and timeframe for reporting adverse events across study sites such that there is a plan in place to report events to and from satellite sites in a timely fashion.

### Special issues

The increasing use of veterinary patients with spontaneous disease as models for translational therapeutic development, as well as the growing trend toward multi-center veterinary clinical studies to improve the pace and quality of research, raise several special issues that require consideration by veterinary boards during study review and approval. These include oversight of satellite locations for study-related activities, the use of healthy animal subjects in research, and the growing need for robust informed consent processes that extend beyond a simple written consent form.

#### Satellite sites

Multi-institutional veterinary clinical studies are growing in frequency, with the goal of conducting more efficient studies that can answer veterinary health questions in a timely manner. With that surge comes the consideration for how satellite sites are handled during study review and approval. This review process should include documentation of information including point of contact, general information about the facility itself, and address. Satellite site participation should be overseen by a licensed veterinarian who has or can establish a valid veterinary-client-patient relationship prior to subject enrollment, will maintain regular communication with the principal investigator/head study coordinator and can serve as a study monitor for the site. Satellite sites should also provide a letter of assurance that they will use a standard consent form approved for use in the study and will adhere to the study’s predetermined protocol. Additionally, for studies with funding from the National Institutes of Health (NIH), satellite sites must be part of the institutional assurance issued to the granting institute. Lastly, the use of unapproved drugs may involve additional stipulations for satellite sites that should be carefully considered to ensure regulatory continuity.

#### Use of healthy animals in research

The scenario of healthy veterinary subjects in research is worth particular consideration, as in this scenario there is often minimal or negligible health benefit for participation, and participation may carry some risk to the individual. Often, healthy veterinary subjects are recruited to participate in studies to serve as a control population with which to compare subjects with a particular disease process, to contribute samples for control or banking purposes, or as part of phase I studies evaluating pharmacokinetics of novel therapies [31]. Considerations for review and approval of healthy-subjects research protocols should be influenced by the level or risk to the participating veterinary patient. Research not involving greater than minimal risk is defined, in the context of the human regulatory perspective, as that for which “the probability and magnitude of harm or discomfort anticipated in the research are not greater in and of themselves than those ordinarily encountered in daily life or during the performance of routine physical or psychological examinations or tests.” [6] While most healthy-subjects research meets these criteria, there are notable examples of studies using healthy human volunteers where adverse events were greater than anticipated, up to and including death [31–33]. As such, phase I studies in healthy animals may involve more than minimal risk, or the risk may not be truly known at the time of study initiation. Review boards should ensure that the consent form clearly delineates what is and is not known about novel therapies that have not been previously administered to the species being studied and that potential impact or patient benefit of the novel therapy is not inflated.

## Conclusions

This document represents a collaborative effort by COHA member institutions to define best practices for veterinary clinical study review and approval by institutional review boards and to highlight areas of need for resource development. To date, site-specific protocols for approval and monitoring have made harmonizing cross-institutional efforts challenging and have created inefficiencies and inconsistencies that decrease rigor and reproducibility and increase time to veterinary clinical study completion. Reconciling local institutional approaches to veterinary clinical study review and implementation represents a first step in leveraging existing COHA resources to create a singular process for veterinary multicenter study review and implementation. This would parallel recent successful efforts with human IRBs to improve efficiency and rigor of veterinary clinical study review, conduct, and ultimate use in both veterinary and translational health research efforts.

As noted throughout, little veterinary-specific literature exists relating to review board assessment of key elements such as risk/benefit, societal value, healthy subjects research, or the consenting process. As the veterinary literature grows in the coming years, reassessment of these guidelines will be key to ensuring continued ethical conduct of veterinary clinical studies that can have an important positive health impact for both veterinary patients, and in many cases, can inform translational knowledge and human health. Additional initiatives may also build on this process by developing other guidelines for veterinary clinical studies, including go/no go criteria associated with adverse event reporting and review and specific processes for post-approval monitoring.

## Data Availability

Data sharing is not applicable to this article as no datasets were generated or analysed during the current study.

## References

[1] Silverman J (2009). Clinical study or research activity?. Lab Animal.

[2] Page R, Baneux P, Vail D (2016). Conduct, oversight, and ethical considerations of clinical trials in companion animals with cancer: report of a workshop on best practice recommendations. J Vet Intern Med.

[3] Kendall LV, Petervary N, Bergdall VK, Page RL, Baneux PJR (2018). Institutional animal care and use committee review of clinical studies. J Am Vet Med Assoc.

[4] National Institutes of Health, Office of Laboratory Animal Welfare. PHS policy on humane care and use of laboratory animals. https://olaw.nih.gov/policies-laws/phs-policy.htm Web site. . Accessed 1 Apr 2020.

[5] Fleischman AR (2016). Ethical issues in neonatal research involving human subjects. Semin Perinatol.

[6] Rossi J, Nelson RM (2017). Minimal risk in pediatric research: a philosophical review and reconsideration. Accountability Res.

[7] Habets MGJL, van Delden JJM, Bredenoord AL (2014). The social value of clinical research. BMC Med Ethics.

[8] Ganguli-Mitra A, Dove ES, Laurie GT, Taylor-Alexander S (2017). Reconfiguring social value in health research through the lens of liminality. Bioethics..

[9] Lutge E, Slack C, Wassenaar D (2017). Defining and negotiating the social value of research in public health facilities: perceptions of stakeholders in a research-active province of South Africa. Bioethics..

[10] Chan A, Tetzlaff JM, Altman DG (2013). SPIRIT 2013 statement: defining standard protocol items for clinical trials. Ann Intern Med.

[11] Kol A, Arzi B, Athanasiou KA (2015). Companion animals: Translational scientist’s new best friends. Sci Transl Med.

[12] Binik A, Hey SP (2019). A framework for assessing scientific merit in ethical review of clinical research. Ethics Human Res.

[13] The ethics police? (2015). The struggle to make human research safe.

[14] Burris S, Moss K (2006). U. S. health researchers review their ethics review boards: a qualitative study. J Empir Res Hum Res Ethics..

[15] Humphreys S, Thomas H, Martin R (2015). Science review in research ethics committees: double jeopardy?: research. Ethics..

[16] Dawson AJ, Yentis SM (2007). Contesting the science/ethics distinction in the review of clinical research. J Med Ethics.

[17] Parkinson B, Meacock R, Sutton M (2019). Designing and using incentives to support recruitment and retention in clinical trials: a scoping review and a checklist for design. Trials..

[18] Brueton VC, Tierney JF, Stenning S (2014). Strategies to improve retention in randomised trials: a cochrane systematic review and meta-analysis. BMJ Open.

[19] Wilkinson M, Moore A (1997). Inducement in research. Bioethics..

[20] Resnik DB (2015). Bioethical issues in providing financial incentives to research participants. Medicoleg Bioeth.

[21] King NM (2000). Defining and describing benefit appropriately in clinical trials. J Law Med Ethics.

[22] Bentley JP, Thacker PG (2004). The influence of risk and monetary payment on the research participation decision making process. J Med Ethics.

[23] Halpern SD, Karlawish JHT, Casarett D, Berlin JA, Asch DA (2004). Empirical assessment of whether moderate payments are undue or unjust inducements for participation in clinical trials. Arch Intern Med.

[24] Singer E, Couper MP (2008). Do incentives exert undue influence on survey participation? experimental evidence. J Empir Res Hum Res Ethics.

[25] Sobolewski J, Bryan JN, Duval D (2019). Readability of consent forms in veterinary clinical research. J Vet Intern Med.

[26] Alahmad G (2018). Informed consent in pediatric oncology: a systematic review of qualitative literature. Cancer Control.

[27] Largent EA, Grady C, Miller FG, Wertheimer A (2012). Money, coercion, and undue inducement: a survey of attitudes about payments to research participants. IRB..

[28] Bracken-Roche D, Bell E, Macdonald ME, Racine E (2017). The concept of ‘vulnerability’ in research ethics: an in-depth analysis of policies and guidelines. Health Res Policy Sys.

[29] Dekking SA, van der Graaf R, van Delden JJ (2014). Strengths and weaknesses of guideline approaches to safeguard voluntary informed consent of patients within a dependent relationship. BMC Med.

[30] Yeates J, Main D (2009). Assessment of companion animal quality of life in veterinary practice and research. J Small Anim Pract.

[31] Karakunnel JJ, Bui N, Palaniappan L (2018). Reviewing the role of healthy volunteer studies in drug development. J Transl Med.

[32] Suntharalingam G, Perry MR, Ward S (2006). Cytokine storm in a phase 1 trial of the anti-CD28 monoclonal antibody TGN1412. N Engl J Med.

[33] Butler D, Callaway E (2016). Scientists in the dark after french clinical trial proves fatal. Nature..

